# Protocol to search for genetic factors related to severe COVID-19 by analyzing publicly available genome-wide association studies

**DOI:** 10.1016/j.xpro.2024.103028

**Published:** 2024-07-30

**Authors:** Ke Zhang, Siyu Lin, Yu-si Luo, Zhongshan Cheng

**Affiliations:** 1 The Key and Characteristic Laboratory of Modern Pathogenicity Biology, School of Basic Medical Sciences, Guizhou Medical University, Guiyang 550025, China; 2 Department of Emergency ICU, The Affiliated Hospital of Guizhou Medical University, Guiyang 550004, China; 3 Center for Applied Bioinformatics, St. Jude Children’s Research Hospital, 262 Danny Thomas Pl, Memphis, TN 38105, USA

**Keywords:** Bioinformatics, Health Sciences, Genetics

## Abstract

COVID-19 casualties vary among different ancestral groups due to a variety of factors. Here, we present a protocol for analyzing publicly available genome-wide association studies (GWASs) to search for ancestry-specific genetic factors related to severe COVID-19. We describe steps for downloading and comparing two COVID-19 GWASs, calculating expression quantitative trait loci, and single-cell gene expression analysis. We demonstrate this approach using GWASs from Host Genetics Initiative; however, it is applicable to other databases such as the UK Biobank.

For complete details on the use and execution of this protocol, please refer to Cheng et al.^1^

## Highlights

• Analyze publicly available COVID-19 genome-wide association studies with SAS
• Compare association signals among GWASs with stack Manhattan plots
• Expression quantitative trait locus analysis with data from the GTEx database
• Differentiate the expression of candidate genes across multiple single-cell types

## Abstract (editor highlights)

Publisher’s note: Undertaking any experimental protocol requires adherence to local institutional guidelines for laboratory safety and ethics.

## Graphical abstract

**Figure:**
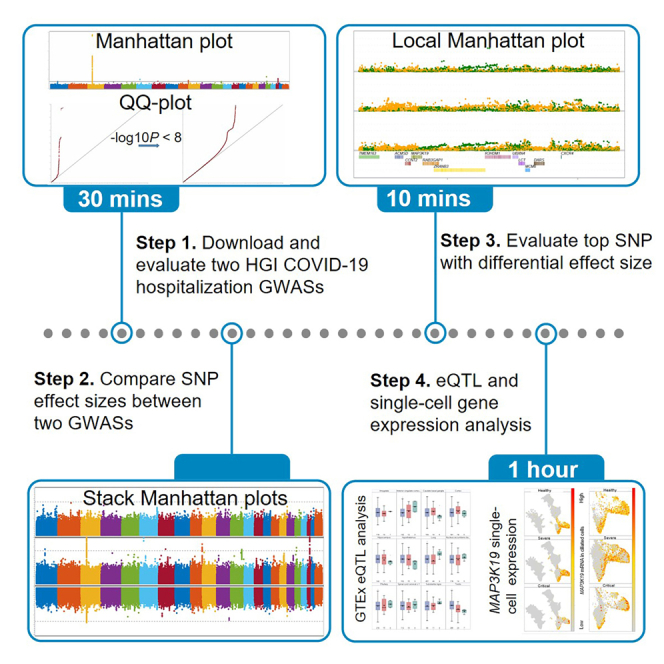

## Before you begin

The protocol below describes the specific steps for using COVID19_GWAS_Analyzer to conduct integrative analyses of COVID-19 hospitalization GWASs from the Host Genetics Initiative (HGI).^2^ However, we have also used this protocol for analyzing GWASs from COVID-19 GRASP database,^3^ UK Biobank (UKB),^4^ and even users’ supplied GWAS data, such as long COVID GWAS data.^5^^,^^6^

To access the necessary tools, register for a free SAS OnDemand for Academics account by visiting this link (https://www.sas.com/en_us/software/on-demand-for-academics.html). SAS OnDemand for Academics provides cloud-based access to SAS Studio free of charge. The current protocol is tailored for execution in the SAS studio.

Once you have obtained a SAS OnDemand for Academics account, download the COVID19_GWAS_Analyzer package in compressed zip format from its corresponding GitHub repository (https://github.com/chengzhongshan/COVID19_GWAS_Analyzer). In SAS Studio, create a directory called ‘Macros’ under the home directory ‘Files (HOME)’. Extract the downloaded file ‘COVID19_GWAS_Analyzer.zip’ on your local computer and upload all SAS macro files within the ‘Macros’ directory to the corresponding online SAS studio directory ‘Macros’.

### Load SAS macros for preparation of analyses

**Timing: ∼2 mins**

1. Log in to SAS studio using SAS OnDemand for Academics.
  a. Press the F4 on your keyboard to create a new SAS file in SAS Studio.
  b. Type or copy the following codes into the SAS file for importing the provided SAS macros in the' Macros' directory located under the SAS Studio home directory.

%let macrodir=%sysfunc(pathname(HOME))/Macros;

%include “&macrodir/importallmacros_ue.sas”;

%importallmacros_ue;

***Note:*** These commands are necessary at the beginning of any new SAS file that utilizes SAS macros from COVID19_GWAS_Analyzer.

2. Execute the above codes by selecting them with your mouse and pressing F3 on the keyboard to submit them to SAS Studio.

**CRITICAL:** Loading SAS macros is a crucial step to enable comprehensive analysis of GWAS data in SAS studio. Even loading > 400 specifically written SAS macros from COVID19_GWAS_Analyzer can be done quickly. To further assist users to know the functions of these SAS macros, we have provided a macro called “get_anno4macro” to print functional annotations of all these macros, and users can read the contents of each macro by providing the macro name to another macro “macroparas”. The full annotation of these SAS macros are deposited into a csv file here: https://github.com/chengzhongshan/COVID19_GWAS_Analyzer/blob/main/Macros/Available_SAS_Macros_and_its_annotations4STAR_PROTOCOL.csv.

## Key resources table

**Table:** 

REAGENT or RESOURCE	SOURCE	IDENTIFIER
**Deposited data**		

The COVID-19 Host Genetics Initiative (HGI) hospitalization GWAS B1 (hospitalized vs. non-hospitalized COVID-19; release 4)	GRASP^3^ and HGI^2^	https://grasp.nhlbi.nih.gov/downloads/COVID19GWAS/10202020/COVID19_HGI_B1_ALL_20201020.b37.txt.gz
The COVID-19 HGI hospitalization GWAS B2 (hospitalized COVID-19 vs. general population; release 4)	GRASP^3^ and HGI^2^	https://grasp.nhlbi.nih.gov/downloads/COVID19GWAS/10202020/COVID19_HGI_B2_ALL_leave_23andme_20201020.b37.txt.gz
Processed differential GWAS summary statistics between HGI-B1 and HGI-B2	This study	https://data.mendeley.com/datasets/7bgym75bjx/1
COVID-19 nasopharyngeal single-cell RNA-seq	Trump et al., 2021^7^	https://cells.ucsc.edu/covid-hypertension/exprMatrix.tsv.gz; https://cells.ucsc.edu/covid-hypertension/meta.tsv; https://cells.ucsc.edu/covid-hypertension/Seurat_umap.coords.tsv.gz

**Software and algorithms**		

SAS OnDemand for Academics	SAS Institute Inc.	https://www.sas.com/en_us/software/on-demand-for-academics.html
UCSC Cell Browser	Speir ML et al.^8^	https://cells.ucsc.edu/
GTEx Portal (version 8)	The GTEx Consortium^9^	https://gtexportal.org/home/
COVID19_GWAS_Analyzer	This study	https://github.com/chengzhongshan/COVID19_GWAS_Analyzer; https://zenodo.org/records/10702503

## Step-by-step method details

### Download and compare two COVID-19 GWASs

**Timing: 50 min**

COVID19_GWAS_Analyzer can carry out differential association analysis to detect SNPs that may have distinct associations with COVID-19 due to ancestry effects or other unobserved factors.

1. Submit the specified codes to SAS Studio to download the two COVID-19 GWASs stored in the GRASP database. Additionally, conduct the comparison of effect sizes for each SNP, the generation of Manhattan plots, and Q-Q plots for both GWASs and the differential GWAS.

∗If the URLs for these GWAS summary statistics were changed or broken in the future, the user can download the two GWAS summary statistics into a local computer and upload them into the HOME directory in SAS OnDemand for Academics;

∗then update the following two URLs as follows;

∗gwas1=%sysfunc(pathname(HOME))/COVID19_HGI_B1_ALL_20201020.b37.txt.gz;

∗gwas2=%sysfunc(pathname(HOME))/COVID19_HGI_B2_ALL_leave_23andme_20201020.b37.txt.gz;

%GRASP_COVID_Hosp_GWAS_Comparison(

gwas1=https://grasp.nhlbi.nih.gov/downloads/COVID19GWAS/10202020/COVID19_HGI_B1_ALL_20201020.b37.txt.gz,

gwas2=https://grasp.nhlbi.nih.gov/downloads/COVID19GWAS/10202020/COVID19_HGI_B2_ALL_leave_23andme_20201020.b37.txt.gz,

outdir=%sysfunc(pathname(HOME)),

mk_manhattan_qqplots4twoGWASs=1

);

2. Three SAS datasets, named 'GWAS1′, 'GWAS2′, and 'GWAS1_vs_2′, will be saved in the home directory of SAS Studio. These datasets can be used in the future to generate Manhattan plots for multiple GWASs or to create local Manhattan plots for the top SNPs.
3. Run the following codes to make a composite Manhattan plot for the generated 3 GWAS datasets above.

libname D “%sysfunc(pathname(HOME))”;

%Manhattan4DiffGWASs(

dsdin=D.GWAS1_vs_2,

pos_var=pos,

chr_var=chr,

P_var=GWAS1_P,

Other_P_vars=GWAS2_P Pval

);

4. Submit the following commands to generate a local Manhattan plot for the top SNP rs16831827 that is close to the gene *MAP3K19.*

libname D “%sysfunc(pathname(HOME))”;

∗It is only needed to run once for importing the hg19 GTF file from gencode;

%let gtf_gz_url=https://ftp.ebi.ac.uk/pub/databases/gencode/Gencode_human/release_19/gencode.v19.annotation.gtf.gz;

%get_genecode_gft_data(

gtf_gz_url=&gtf_gz_url,

outdsd=D.GTF_HG19

);

∗Previously generated SAS dataset GWAS1_vs_2 is stored in the SAS library ‘D’;

%SNP_Local_Manhattan_With_GTF(

gwas_dsd=D.GWAS1_vs_2,

chr_var=chr,

AssocPVars=GWAS1_P GWAS2_P pval,

SNP_IDs=rs16831827,

/∗if providing chr:pos or chr:st:end, it will query by positions ranging from start to end positions on the specific chromosome!∗/

SNP_Var=rsid,

Pos_Var=pos,

gtf_dsd=D.GTF_HG19,

ZscoreVars=GWAS1_z GWAS2_z diff_zscore,

/∗Can be beta1 beat2 or other numeric vars indicating assoc or other +/- directions∗/

gwas_labels_in_order=HGI_B1 HGI_B2 HGI_B1_vs_B2,

design_width=1300,

design_height=750

);

**CRITICAL:** The two compressed GWAS summary statistics were generated by HGI and shared via the GRASP database. COVID19_GWAS_Analyzer can efficiently download and process compressed data, saving both space and time.

5. Use HaploReg 4.2^10^ to evaluate the minor allele frequency (MAF) of rs16831827 across multiple populations.
6. Confirm the significant difference of rs16831827 MAF between African population and other populations, including populations from East Asian, South Asian, Ad Mixed American, and Europe (https://pubs.broadinstitute.org/mammals/haploreg/detail_v4.2.php?query=&id=rs16831827).

### Calculate expression quantitative trait locus (eQTL)

**Timing:** ∼1 min

COVID19_GWAS_Analyzer has implemented the calculation of eQTL between any SNP and genes, provided the SNP and target genes are both included in the GTEx database.^9^ This analysis will generate boxplots that visualize the potential correlation between a SNP and a gene across 49 tissues and display the association strength of eQTL in a heatmap based on GTEx API-generated association *p* values. Notably, most top GWAS SNPs are situated in non-coding regions and can act as cis- or trans-eQTLs for genes located nearby or on a different chromosome.

7. Run the SAS macro ‘CaculateMulteQTLs_in_GTEx’ to perform eQTL analysis for rs16831827, which emerges as the top hit in the differential association analysis between the two GWASs, including HGI-B1 and HGI-B2.

%CaculateMulteQTLs_in_GTEx(

query_snps=rs16831827,

gene=MAP3K19,

genoexp_outdsd=geno_exp,

eQTLSumOutdsd=AssocSummary,

create_eqtl_boxplots=1

);

***Note:*** Remember to load all SAS macros before running the calculation of eQTL.

### Single-cell gene expression analysis

**Timing: ∼30 mins**

Once the top GWAS SNPs can be associated with specific genes through the eQTL analysis, it would be worthwhile to investigate further the expression of these genes in biologically related single-cell datasets available from the UCSC Cell Browser.^8^ After locating the desired single-cell dataset and obtaining its corresponding download links, the COVID19_GWAS_Analyzer can be utilized to generate customized Uniform Manifold Approximation and Projection (UMAP) and gene expression figures stratified by sample groups, such as distinct phenotypes of these samples.

8. For demonstration purposes, please download the COVID-19 single-cell dataset published by Trump et al.^7^ by executing the following SAS codes:

∗Download the following single-cell data from UCSC Cell Browser into a local computer and then upload them into the HOME directory of SAS OnDemand for Academics;

∗https://cells.ucsc.edu/covid-hypertension/Seurat_umap.coords.tsv.gz;

∗https://cells.ucsc.edu/covid-hypertension/meta.tsv;

∗https://cells.ucsc.edu/covid-hypertension/exprMatrix.tsv.gz;

%import_sc_mtex_meta_umap_data(

umap_file=%sysfunc(pathname(HOME))/Seurat_umap.coords.tsv.gz,

meta_file=%sysfunc(pathname(HOME))/meta.tsv,

cell_id_in_meta=cell,

exp_matrix_file=%sysfunc(pathname(HOME))/exprMatrix.tsv.gz,

outdir=%sysfunc(pathname(HOME)),

/∗Three sas datasets will be created and put into the dir:

exp (read matrix with column headers, and the last column is for genesymbol),

umap (umap coordinates with sample meta data merged),

headers (cell barcodes corresponding to column headers for the exp matrix)∗/

target_genes=,

/∗Provide genesymbols separated by | for parsing lines match with these genes;

when the single cell dataset is too large, this will save disk space in SAS OnDemand for Academics∗/

mean_exp_cutoff=0.01, /∗Only keep row records with mean read of gene expression > mean_exp_cutoff∗/

max_cells2import=1000000 /∗Maximum number of cells to be imported;

if there are more than 1 million cells in the input cell matrix, only 1 million cells

will be randomly selected.∗/

);

***Note:*** if the total number of cells in the single-cell matrix is more than 1 million, the macro will randomly select 1 million cells automatically, which will avoid using up the limited disk space (∼5 GB) in SAS OnDemand for Academics.

9. Create a single-cell UMAP by entering the following codes into SAS studio.

libname sc "%sysfunc(pathname(HOME))";

%sc_umap(

umap_ds=sc.umap,

xvar=x,

yvar=y,

cluster_var=cluster

);

10. Create a single-cell UMAP visualization for the target gene *MAP3K19*, emphasizing only the major cell types that express *MAP3K19*, specifically ciliated cell types, using the parameter 'where_cnd4sgplot′ for the macro ‘sc_scatter4gene’.

libname sc "%sysfunc(pathname(HOME))";

∗Modify phenotype categories;

data sc.umap;

set sc.umap;

if severity=”healthy_control” then severity=”Healthy”;

if severity=”severe” then severity=”Severe”;

if severity=”critical” then severity=”Critical”;

run;

%sc_scatter4gene(

dsd=sc.exp,

dsd_headers=sc.headers,

dsd_umap=sc.umap,

gene=MAP3K19,

pheno_var=severity,

pheno_categories=Healthy Severe Critical,

boxplot_width=800,

boxplot_height=300,

umap_width=400,

umap_height=800,

umap_lattice_nrows=3,

boxplot_nrows=1,

where_cnd4sgplot=%quote(cluster contains %'Ciliated%')

);

∗Further zoom into specific UMAP area defined by the X- and Y-axis regions for single cells mainly express MAP3K19.;

∗Note: the input SAS dataset ‘new__tgt_dsd_’ is internally generated by the above SAS macro sc_scatter4gene;

%umap_with_axes_restriction(

dsdin=new__tgt_dsd_,

umap_width=1000,

umap_height=300,

lattice_or_not=0,

raxis_max=20000,

raxis_min=0,

caxis_max=,

caxis_min=45000,

panel_row_num=1,

noheader=0

);

11. Analyze the percentage distribution of different cell types in the single-cell dataset.
12. Calculate the proportion of cells expressing *MAP3K19* in each individual cell type.
13. Perform differential gene expression analysis for *MAP3K19* using the SAS procedure proc GLM.

libname sc "%sysfunc(pathname(HOME))";

∗Use the dataset ‘new__tgt_dsd_’ internally generated by running;

∗the SAS macro sc_scatter4gene;

∗The following steps combine non-ciliated cells as ‘Other’ and;

∗treat samples with age>60 as ‘Yes’ and others as ‘No’;

data tgt;

length cell_type $50.;

set new__tgt_dsd_;

cell_type="Other";

if prxmatch('/Ciliated/i',Cluster) then cell_type=Cluster;

if age>60 then Old='Yes';

else Old='No';

run;

∗Get percentages of different cells in the single cell dataset;

∗Note: we use the exp_cutoff=-1; ∗This will calculate the percentages of different cells within each pheno group; ∗percent=cells/total_cells_in_a_pheno_grp; ∗The macro also perform differential gene expression analysis based on log10(reads+1); %sc_freq_boxplot(longformdsd=tgt,cell_type_var=cell_type, sample_grp_var=sample, pheno_var=severity, cust_pheno_order=Healthy Severe Critical, exp_var=exp, exp_cutoff=-1, boxplot_height=800, boxplot_width=300, boxplot_nrows=5, where_cnd_for_sgplot=%quote( cell_type contains 'Ciliated' or cell_type='Other'), frqout=cellfrqout, other_glm_classes=sex medication Old CAD CVD hypertension,

/∗covariant for the linear model∗/

aggre_sc_glm_pdiff_dsd=all_sc ); ∗Note: the differential expression analysis results can be further viewed by running the following code;

proc print data=all_sc_celltype_pdiff;

run;

***Note:*** After initiating the library 'sc', the previously imported single-cell SAS datasets will be accessible to the SAS macros. The final differential gene expression results will be saved into a SAS table named as 'all_sc_celltype_pdiff'.

## Expected outcomes

**Figure 1:**
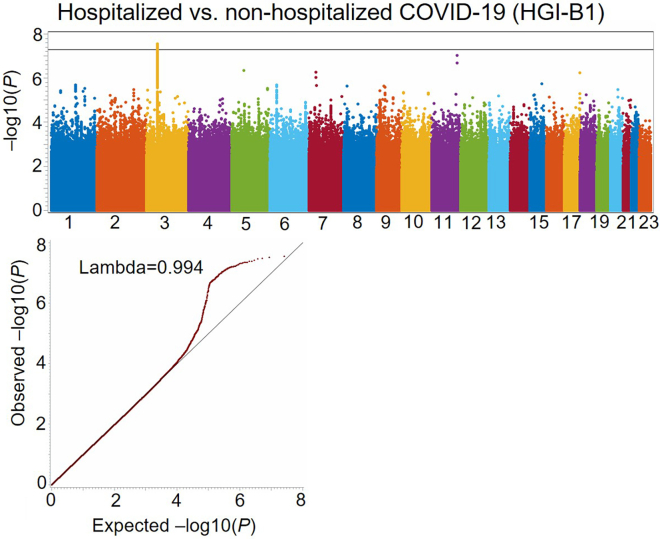
Manhattan plot and Q-Q plots for the HGI COVID-19 hospitalization GWAS (HGI-B1) comparing hospitalized and non-hospitalized COVID-19 cases with mixed ancestries

**Figure 2:**
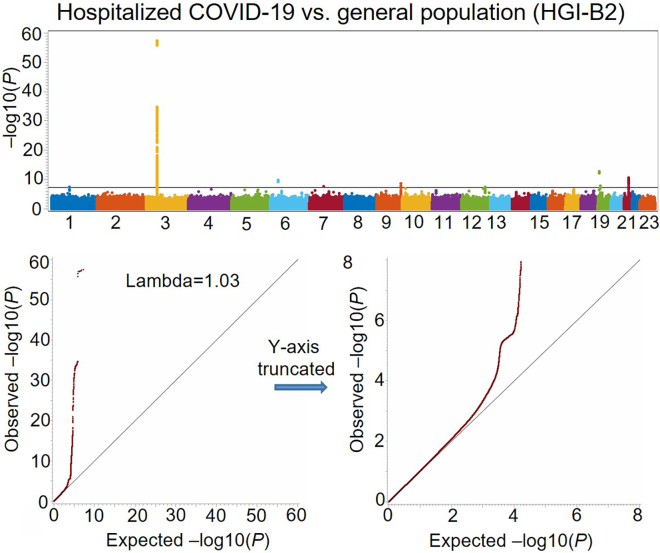
Manhattan plot and Q-Q plots for the COVID-19 hospitalization GWAS (HGI-B1) comparing hospitalized COVID-19 cases with general population controls with mixed ancestries

**Figure 3:**
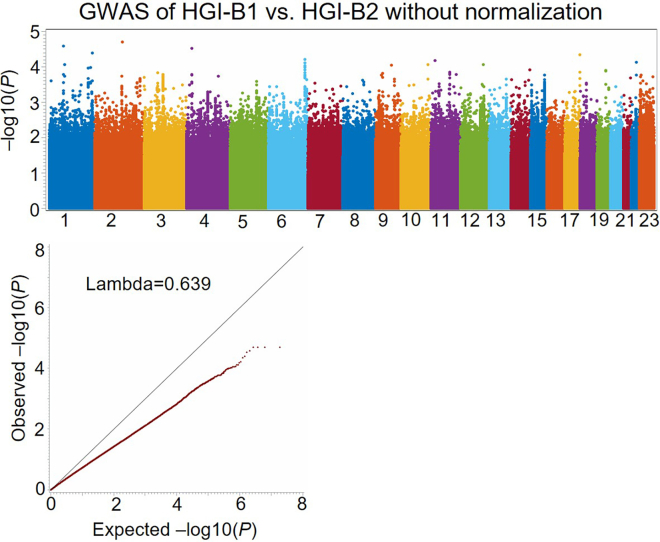
Manhattan plot and Q-Q plots for unadjusted differential association signals, not taking account of overlapping samples between HGI-B1 and HGI-B2 GWASs The deflation of differential association signals between the two GWASs is due to the overlapping samples used by the two GWASs.

**Figure 4:**
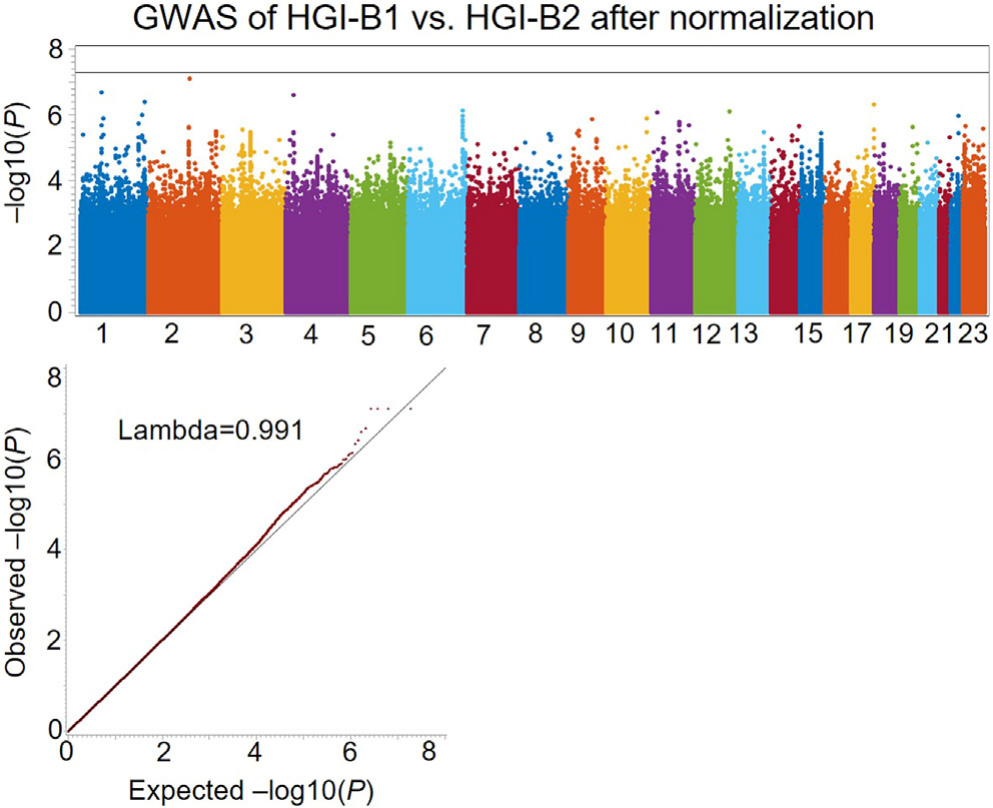
Manhattan plot and Q-Q plots for normalized differential association signals accounting for overlapping samples between HGI-B1 and HGI-B2 GWASs No obvious inflation or deflation is observed in the differential association signals after normalization.

**Figure 5:**
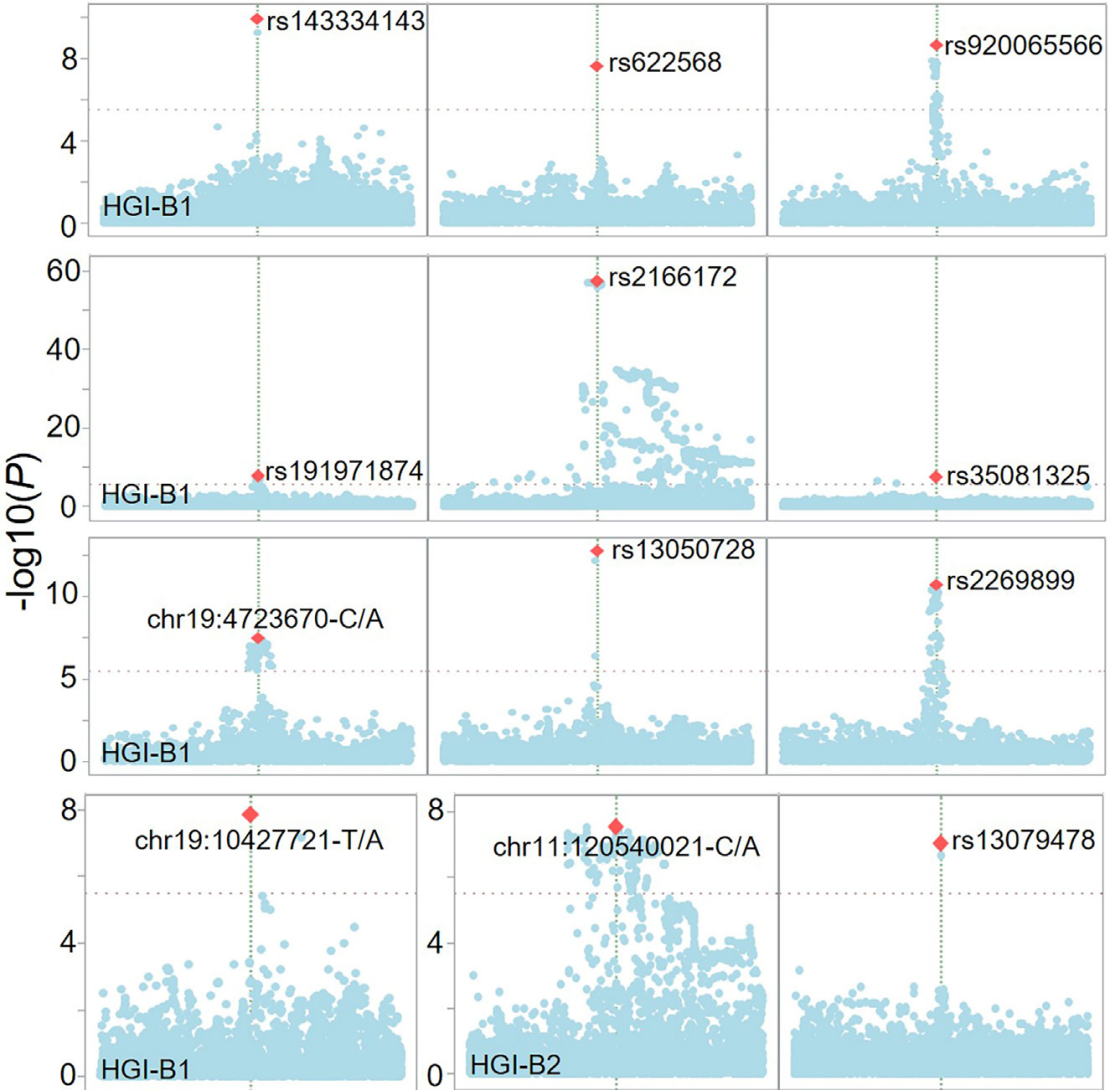
Top independent SNPs from HGI-B1 and HGI-B2 GWASs shown in local Manhattan plots Red diamond indicates the SNP with the most significant association centered in a 1-Mb window.

After running the SAS macro 'GRASP_COVID_Hosp_GWAS_Comparison', Manhattan plots and Q-Q plots for four GWASs, including HGI-B1, HGI-B2, unadjusted HGI-B1 vs. HGI-B2, and normalized HGI-B1 vs. HGI-B2, will be created as shown in Figures 1, 2, 3, and 4. For the top SNPs that passed the threshold of *P* < 1×10^−7^ in the two GWASs, including HGI-B1 and HGI-B2, local Manhattan plots of these SNPs will be generated around a 1 Mb-window where each of these SNPs is located at the center (see Figure 5).

**Figure 6:**
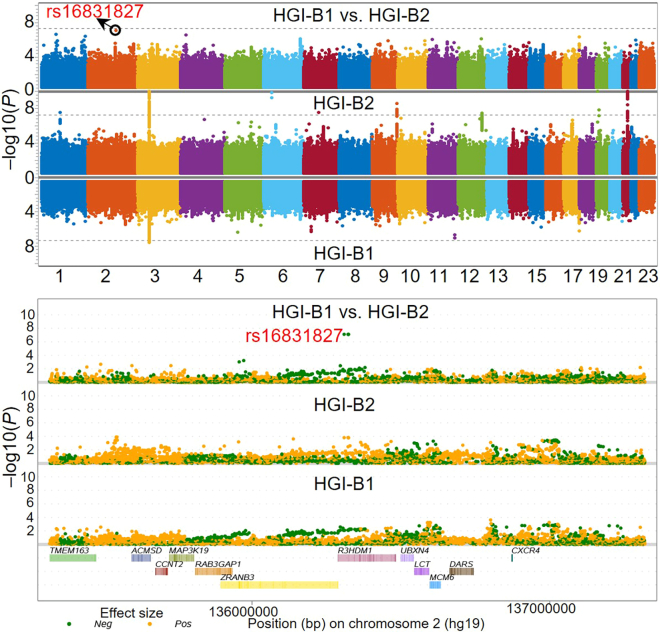
Comparison of the two GWASs, including HGI-B1 and HGI-B2, using Manhattan plots The differential association signals between the two GWASs are illustrated at the top and a local Manhattan plot for the top SNP showing differential effect sizes between the two GWASs is displayed at the bottom.

Two SAS macros, 'Manhattan4DiffGWASs' and 'SNP_Local_Manhattan_With_GTF', will display GWAS association signals from HGI-B1, HGI-B2, and the normalized HGI-B1 vs. HGI-B2, in a compact Manhattan plot or a local Manhattan plot. The top SNP rs16831827, with differential effect sizes between HGI-B1 and HGI-B2, will be further illustrated for association signals around it in a local Manhattan plot, including association signals from the above 3 GWASs (see Figure 6).

**Figure 7:**
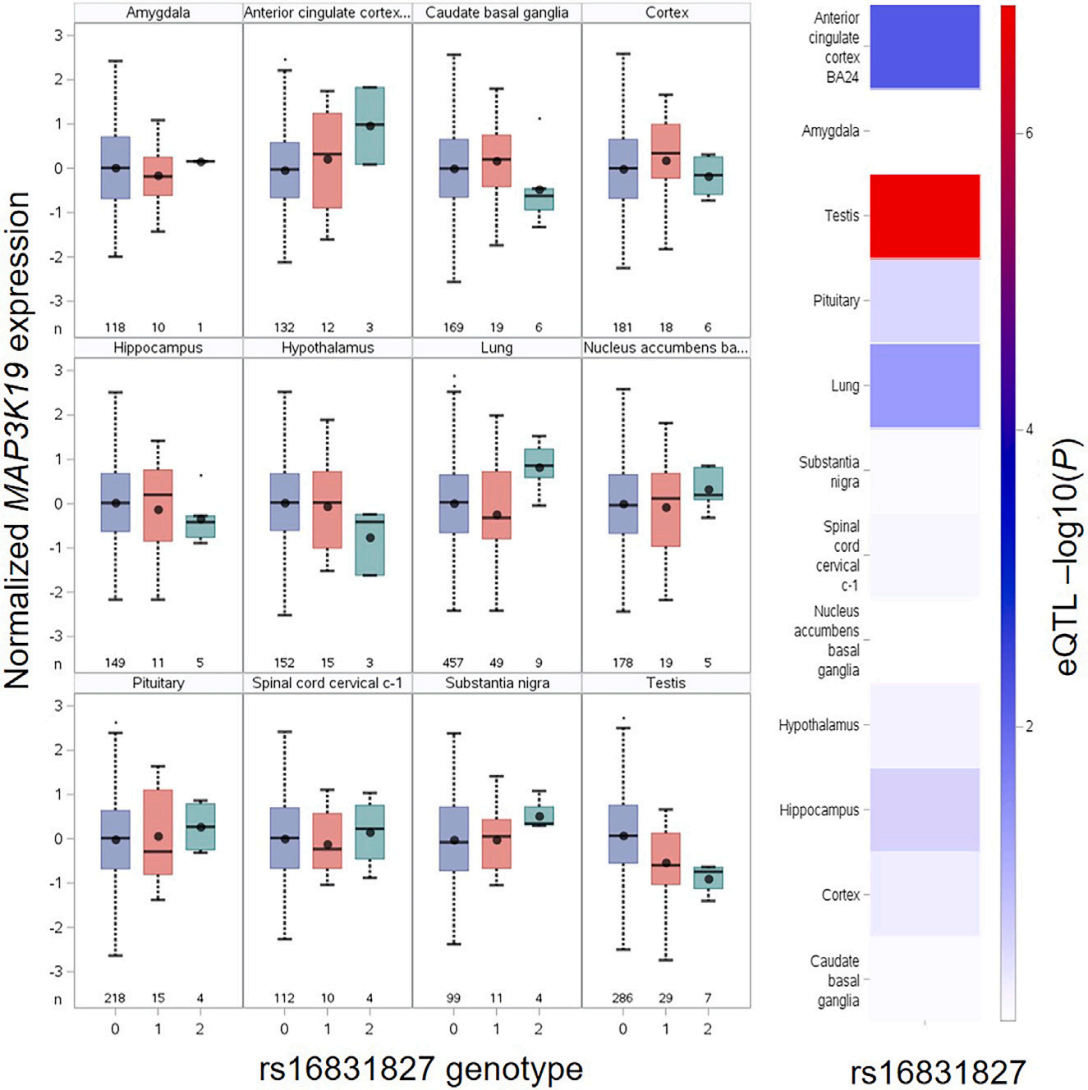
Expression quantitative trait locus (eQTL) analysis for the COVID-19 risk single nucleotide polymorphism rs16831827 based on GTEx data Boxplots demonstrate the correlation between the rs16831827 genotype and *MAP3K19* across 16 GTEx tissues with detectable *MAP3K19* expression. The eQTL -log10(*P*) values are displayed in the heatmap located on the right panel. Boxplot is utilized to demonstration the eQTL results with descriptive statistics, including mean (dot), median (a horizontal line within the box), the interquartile range (IQR; box region), minimum (a horizontal line attached to the lower dashed line), maximum (a horizontal line attached to the upper dashed line), and outliers represented by dot outside of box, as well as the variability of the minimum, maximum illustrated by the two whiskers.

The SAS macro 'CaculateMulteQTLs_in_GTEx' will create eQTL boxplots displaying the correlations between rs16831827 and *MAP3K19* expression across GTEx tissues with detectable expression of *MAP3K19*, as shown in Figure 7.

**Figure 8:**
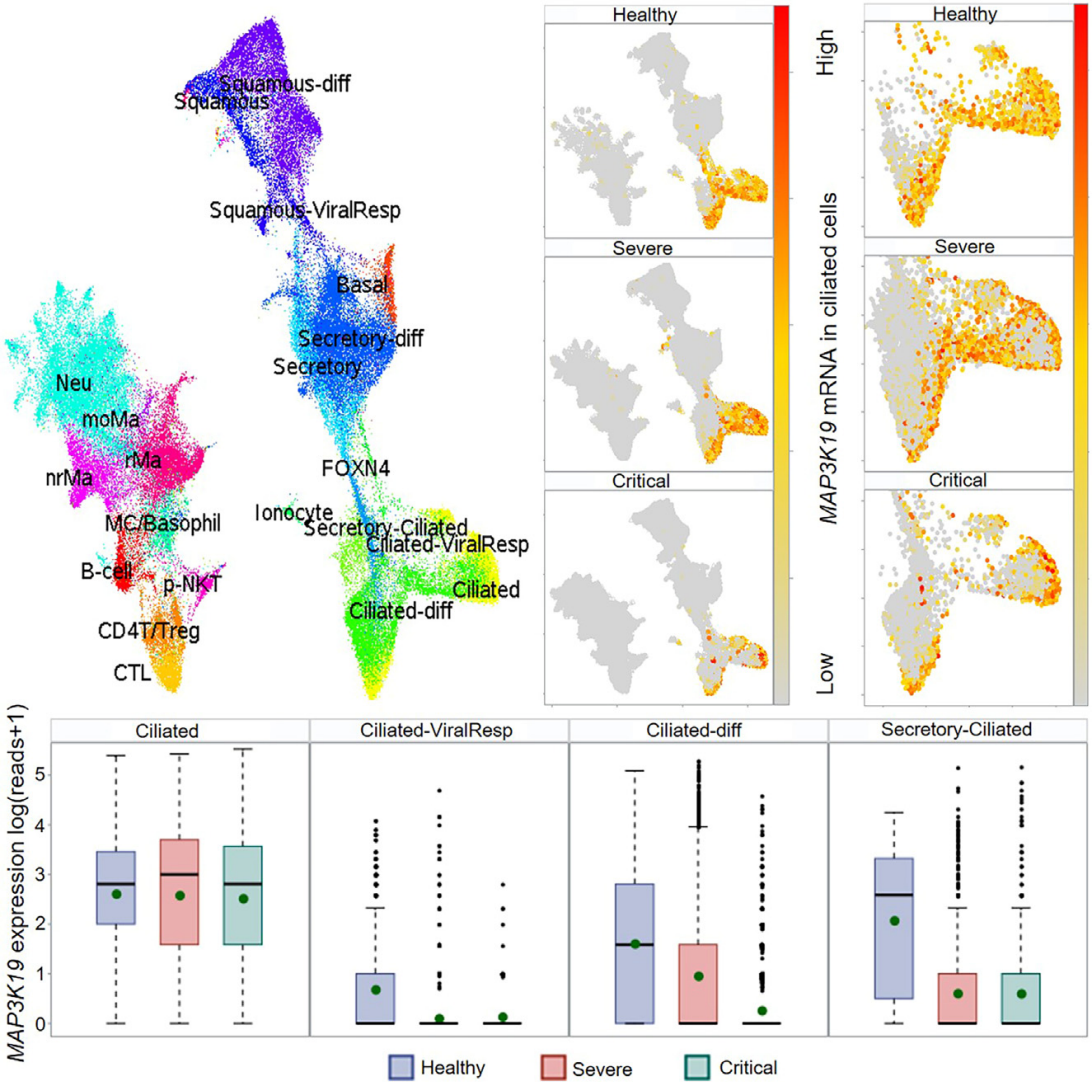
Single-cell expression analysis of *MAP3K19* among nasopharyngeal samples of severe (*n* = 23), critical (*n* = 9) COVID-19 patients and healthy controls (*n* = 16) Uniform Manifold Approximation and Projections (UMAPs) illustrate that *MAP3K19* is mainly expressed in ciliated cell types, and boxplots demonstrate reduced *MAP3K19* expression correlated with increased COVID-19 severity. Boxplot displays descriptive statistics, including mean (dot), median (a horizontal line within the box), the interquartile range (IQR; box region), minimum (a horizontal line attached to the lower dashed line), maximum (a horizontal line attached to the upper dashed line), and outliers represented by dot outside of the box, as well as the variability of the minimum, maximum illustrated by the two whiskers.

Regarding single-cell expression analysis for *MAP3K19*, COVID19_GWAS_Aanlyzer will employ several SAS macros to visualize the specific expression of *MAP3K19* in ciliated cell types across different samples, such as healthy controls, severe COVID-19 patients, and critical COVID-19 patients (see Figure 8). Differential gene expression analysis is also performed across each ciliated cell type for *MAP3K19* by using the SAS macro 'sc_freq_boxplot', with the results stored in a SAS dataset 'all_sc_celltype_pdiff', which can be printed using proc print procedure in SAS Studio.

## Quantification and statistical analysis

1. To calculate differential effect sizes between two GWASs, the differential Z-test^11^^,^^12^ is employed to compare the effect sizes of each common SNP (MAF > 0.01, imputation score > 0.6) between the two GWASs. The test considers the possibility of normalizing the effect of overlapped samples between HGI-B1 and HGI-B2 on the estimated variance, i.e., gwas1.se^2^ + gwas2.se^2^.
2. To calculate the differential effect size of each SNP from HGI-B1 and HGI-B2 without normalization, users can follow the procedure as outlined in Sentence 1 using the following formulas:

$$\Delta Z-\text{score}=\frac{\text{gwas}1.\beta -\text{gwas}2.\beta }{\sqrt{\left({\text{gwas}1.\text{se}}^{2}+\text{gwas}2.{\text{se}}^{2}\right)}}$$

diff.zscore=ΔZ−score

P=pnorm(−|diff.zscore|)∗2

3. To calculate the differential effect size of each SNP from HGI-B1 and HGI-B2 with normalization, COVID19_GWAS_Analyzer will first normalize the raw differential Z-score using SAS procedure proc stdize with the method ‘std’, and then proceed with the differential *p* value calculation using normalized data.

$$\Delta Z-\text{score}=\frac{\text{gwas}1.\beta -\text{gwas}2.\beta }{\sqrt{\left({\text{gwas}1.\text{se}}^{2}+\text{gwas}2.{\text{se}}^{2}\right)}}$$

diff.zscore=Normalization(ΔZ−score)

P=pnorm(−|diff.zscore|)∗2

## Limitations

The COVID19_GWAS_Analyzer has been implemented using the SAS statistical language and successfully tested in the SAS Studio of SAS OnDemand for Academics—a cloud-computing platform pre-installed with SAS, accessible via any web browser. This platform is freely available to users lacking sufficient computing resources for data analysis. Our goal is to leverage SAS OnDemand for Academics for exploratory data analysis on extensive COVID-19 datasets available on the internet, encompassing COVID-19 GWAS summary statistics, expression quantitative trait locus data, and single-cell expression data. While SAS OnDemand for Academics provides ∼30 GB of memory for computation, the limited 5 GB disk space for data storage can pose challenges, especially when analyzing GWAS or processing single-cell data. To address this, we limit the total number of input cells to a modifiable threshold, such as no more than 1 million, or the users can query only candidate genes. To overcome disk space constraints when analyzing multiple large GWAS datasets, users can focus on SNPs with a higher MAF, such as MAF > 0.01, and imputation quality score > 0.6. For merging summary statistics from more than 2 GWASs, we recommend using our custom Perl script available on GitHub on a local computer of Windows or Linux system with Perl installed (https://github.com/chengzhongshan/COVID19_GWAS_Analyzer/blob/main/LongCOVID_data_and_scripts/MergeBigFiles.PL). This script combines selected columns of GWASs, of which the resulting file after compression using 7Zip can then be uploaded to SAS OnDemand for Academics for further analysis. When dealing with extensive single-cell data, it is advisable to focus on specific genes, such as those with mean read counts > 0.01, or randomly select fewer than 1 million cells during data importation using the SAS macro 'import_sc_mtex_meta_umap_data'. Alternatively, the COVID19_GWAS_Analyzer can be executed on a local computer or in a cloud environment if users have access to the SAS9.4 workbench for Windows or Linux systems, in which there should be no issues for the 5 GB disk space limitation as mentioned above.

While the COVID19_GWAS_Analyzer is capable of downloading GWAS data from public databases such as COVID-19 HGI, GRASP, and UK Biobank, the majority of its included macros can be tailored for the analysis of GWAS or single-cell data from alternative sources, including users' own datasets. Even users lacking basic coding skills in the SAS statistical language can effectively utilize these SAS macros and combine them for various purposes. With over 400 SAS macros within COVID19_GWAS_Analyzer, we offer two simplified macros, "get_anno4macro" and "macroparas", to assist researchers in quickly understanding the functionalities of these macros. For instance, a crucial SAS macro, "ImportFileHeaderFromZIP", enables the importation of uncompressed or compressed files into SAS Studio, with flexible settings allowing users to select specific columns for data importation. In our testing, a compressed single-cell expression matrix (∼1 GB), equivalent to ∼9 GB in uncompressed mode and containing approximately 135,000 single cells, can be efficiently processed by the SAS macro "ucsc_cell_matrix2wideformatdsd". Furthermore, the SAS macro "ucsc_cell_matrix2wideformatdsd" will randomly select 1 million cells if the input single-cell expression matrix contains more than 1 million cells, mitigating the risk of exceeding the limited 5 GB disk space in SAS OnDemand for Academics. Additionally, the 'Tasks and Utilities → Utilities → Import Data' function in SAS Studio facilitates the importation of uncompressed text files without requiring coding. Once GWAS summary statistics or single-cell datasets are imported into SAS Studio, the 'Tasks and Utilities → Utilities → Query' function provided by SAS Studio can be employed for data filtering and other statistical analyses, eliminating the need for coding skills.

Please be aware that the functionalities of COVID19_GWAS_Analyzer are tailored specifically for the visualization of COVID-19-related GWAS data, for biologists or geneticists interested in exploring candidate genes potentially implicated in COVID-19. The tool is created to showcase COVID-19 association signals across multiple GWASs, focusing on COVID-19-related phenotypes or other disease traits through a stacked local Manhattan plot. This visual representation allows for direct evaluation of potential candidate genes. The incorporation of eQTL analysis and the visualization of single-cell gene expression add depth to the tool, aiding biologists or geneticists in understanding the potential roles played by these candidate genes across different single-cell types or COVID-19 phenotypes. It’s noteworthy that COVID19_GWAS_Analyzer can access GTEx data, providing users with both gene expression data and de-identified genotype data, as well as eQTL boxplots. For interactive viewing of association signals from a single GWAS, users are recommended to utilize the online server of locuszoom.^13^ However, it’s important to note that this server lacks the capability to generate a stacked local Manhattan plot for multiple GWASs. In the case of genome-wide single-cell analysis, the R package Seurat^14^ can be employed to analyze specific single-cell data downloaded from UCSC Cell Browser.^8^ The sctransform function^15^ in Seurat facilitates the normalization and differentiation of single-cell expression patterns on genome-wide among selected COVID-19 single-cell expression data. Given that single-cell differential gene expression is an active research area, users may consider following the suggestions proposed by Lause et al.,^15^ who have evaluated various methods, including traditional T-Test and other model-based approaches for single-cell differential expression analysis. Lastly, COVID19_GWAS_Analyzer proves valuable in visualizing single-cell data inspired by UCSC Cell Browser, and offering additional capability to conduct customized analyses by incorporating extra clinical phenotypes for the single-cell data.

## Troubleshooting

### Problem 1

One possible scenario is that after submitting the codes provided in this manuscript, the SAS studio stops immediately due to an excessive number of errors or warnings, making it too complex to debug (Related to Step 1).

### Potential solution

• When submitting codes in SAS studio, SAS studio will first compile the SAS macros and resolve macro variables with the supplied values. It is advisable to locate the first error shown in the SAS studio log window and determine if the macro variables have the appropriate values assigned to the submitted SAS macro.
• Users can run the command ‘options mprint mlogic symbolgen;’ before submitting previously failed SAS codes. This command will display the resolved values for these macro variables in the SAS studio log window. Users can then pinpoint potential suspicious macro variables and their corresponding values near the initial error.

### Problem 2

The most frequently occurring error encountered when using COVID19_GWAS_Analyzer is the following message that appears in the SAS log window within SAS Studio (Related to Step 2):

“WARNING: Apparent invocation of macro MACRO_NAME not resolved."

### Potential solution

• This error occurred because the macro was not loaded into SAS studio.
• To resolve this issue, you need to import all the macros saved in the 'Macros' directory located under the home directory of SAS studio.

### Problem 3

It is a common practice for users to process their own GWAS or single-cell data. Alternatively, if the download URL links for these data are not accessible online, they can still be processed using COVID19_GWAS_Analyzer. However, the users would be required to upload the data into the SAS OnDemand for Academics, in plain text, compressed zip, or gz file formats (Related to Steps 3 and 10).

### Potential solution

• Compress users' own GWAS or single-cell data using 7-Zip in 'zip' or 'gz' format to save time and space for SAS OnDemand for Academics.
• Create a directory called 'data' under the home directory 'Files (HOME)' of SAS studio. The full path for the newly created directory would be as follows:

%sysfunc(pathname(HOME))/data

• Replace the URL links for the target SAS macros with the full paths of these uploaded files in SAS studio, like the below path:

%sysfunc(pathname(HOME))/data/GWAS.gz

• Run the target SAS macro using users’ own data.

### Problem 4

One common task is to evaluate the SAS dataset generated by a target SAS macro. Since inspecting the output can be beneficial for further analysis, users may need to view the SAS dataset to understand the output better. (Related to Step 3).

### Potential solution

• In SAS studio, users can select the file containing the submitted SAS codes and then use the mouse to click the 'RESULTS' button, which leads to the display of the 'Table' button. Users can select the target SAS dataset for interactive viewing within SAS studio by clicking the' Table' button. Users can filter and sort the table based on their specific criteria.
• If users prefer to use SAS commands to view a specific SAS dataset, the following SAS codes can be modified to print records in the dataset:

proc print data=sas_data_set;

∗Where [input customized filters for specific variables in the SAS data set];

where SNP=”rs16831827”;

run;

### Problem 5

How to verify the header of a compressed 'gz' or 'zip' file uploaded or downloaded in SAS studio? (Related to Step 3).

### Potential solution

• Users typically desire to comprehend the headers of a specific 'gz' file. However, SAS studio doesn’t have simple functions to directly read the compressed 'gz' or 'zip' file, the SAS macro 'check_header_and_values.sas' can effectively print the headers of the compressed file for further analyses.
• After users have obtained the column names within the compressed file, they can employ the SAS macro 'ImportFileHeadersFromZIP' to import raw data into a SAS dataset.

### Problem 6

To use COVID19_GWAS_Analyzer on any additional GWAS datasets from sources such as the COVID-19 GRASP database, UK Biobank, HGI, or single cell datasets from UCSC Cell Browser, the crucial initial step is securing the corresponding URL links. However, the question is how do we obtain these URLs? (Related to Steps 3 and 10).

### Potential solution

• To access the GWAS datasets from GRASP (https://grasp.nhlbi.nih.gov/covid19GWASResults.aspx) and HGI (https://www.covid19hg.org/results/r7; replace the release number 'r2′ to r2-r7 for specific release number), users must visit the provided websites and locate the web pages containing the desired GWAS. They should then right-click their mouse and select "Copy URL" before updating the URL for the SAS macros 'get_HGI_covid_gwas_from_HGI' (for releases < r7), 'get_covid_gwas_from_grasp', and 'get_HGI_R7_GWAS'.
• For all GWASs from the latest release 7 of HGI, users can search for specific SNPs using the SAS macro 'get_sigs_from_hgi_r7_by_chrpos'. This macro includes all GWAS URL links; users only need to select the target GWASs by name.
• To access UK Biobank GWAS, visit the website from Neale’s lab (GWAS round 2: http://www.nealelab.is/uk-biobank). Click on the item 'GWAS round 2 results can be found here' to view the excel sheet 'Manifest 201807′ online in the GWAS Excel table. Then, copy the URL link for the target GWAS and update it for the SAS macro 'get_UKB_gwas_from_UKB'.
• Finally, for single-cell datasets included in the UCSC Cell Browser (https://cells.ucsc.edu/), there are over 200 available single-cell datasets. Users can open the provided website of UCSC Cell Browser and select their desired single-celldata set. When the mouse cursor hovers over an interesting single-cell data set, right-click it to display its description. Here, they will find 'Data Download', where they can copy the URL links for the single-cell expression matrix, UMAP file, and sample meta file. Users can then update these links for the SAS macro 'import_sc_mtex_meta_umap_data' or download these data into a local computer, and then upload them into SAS studio for further analyses.

### Problem 7

Because of the restricted disk space provided by SAS OnDemand for Academics (approximately 5 GB), it may not be possible to merge more than three GWAS datasets using proc sql in SAS studio. How do we merge multiple GWAS datasets (n >= 3) and visualize them with a composite Manhattan plot in SAS studio? (Related to Step 5).

### Potential solution

• Download these target GWAS datasets into a directory in a local computer, uncompress them and delete the original compressed files. Obtain this handy Perl script (https://github.com/chengzhongshan/COVID19_GWAS_Analyzer/tree/main/LongCOVID_data_and_scripts/MergeBigFiles.PL) to merge these GWAS files by selecting essential common columns from the same directory containing these uncompressed GWAS datasets.
• Compress the merged GWAS file into ‘gz’ format using 7Zip and upload it into SAS studio.
• Users can import the compressed GWAS file by running the SAS macro ‘ImportFileHeadersFromZIP’ and visualize the GWAS association signals across multiple GWASs using the two SAS macros, including ‘Manhattan4DiffGWASs’ and ‘SNP_Local_Manhattan_With_GTF’.

### Problem 8

When executing the SAS macro 'sc_freq_boxplot', no output figures or tables were generated, and the following error message appeared in the log window of SAS studio (Related to Steps 13, 14, and 15):

“ERROR: One or more variables are missing, or freq or weight is zero on every observation.”

### Potential solution

• If the input phenotype groups for the parameter 'cust_pheno_order' do not match the phenotype categories in the input dataset, the macro will fail to generate figures and tables due to the above error.
• To resolve this issue, match the names of the phenotype groups in the input dataset precisely with those provided to the parameter 'cust_pheno_order'.

## Resource availability

### Lead contact

Further information and requests for resources and reagents should be directed to and will be fulfilled by the lead contact, Zhongshan Cheng (cheng.zhong.shan@gmail.com).

### Technical contact

Any technical questions will be fulfilled by the Lead contact, Zhongshan Cheng (cheng.zhong.shan@gmail.com).

### Materials availability

All materials used in current protocols are freely available as listed in the Resource table and can be downloaded and analyzed automatically by running the COVID19_GWAS_Analyzer in SAS OnDemand for Academics.

### Data and code availability

All data and codes are included in the COVID19_GWAS_Analyzer shared via GitHub (https://github.com/chengzhongshan/COVID19_GWAS_Analyzer).

• This study does not report new original data.
• Processed data have been deposited to Mendeley Data: https://data.mendeley.com/datasets/7bgym75bjx/1
• Any additional information or requests regarding how to access or analyze the data included in this paper should be directed to the lead contact.
